# Level of health extension service utilization and associated factors among community in Abuna Gindeberet District, West Shoa Zone, Oromia Regional State, Ethiopia

**DOI:** 10.1186/1472-6963-14-324

**Published:** 2014-07-28

**Authors:** Zewudu Kelbessa, Negga Baraki, Gudina Egata

**Affiliations:** 1 Zonal Health office Communicable Disease Prevention and Control Department, P.O. Box: 107, Ambo, Ethiopia; 2 Haramaya University, College of Medicine and Health Science, School of Graduate Study, Harar, Ethiopia

**Keywords:** Model family, Knowledge, Community participation, Health extension services

## Abstract

**Background:**

In Ethiopia, children and mothers have been facing several health problems due to poor access to modern health care facilities and lack of effective demand to utilize the available ones. In response to this, the Ethiopian government initiated the health extension program in 2003 to improve equity in access to preventive, promotive and selected curative health interventions through health extension program. However, the level of health extension service utilization is not known. Therefore, this study presents the level of health extension service utilization and associated factors.

**Methods:**

A community based cross sectional study was carried out from February to March 2012. Data was collected through face-to-face interview by using pretested structured questionnaires adopted from review of different related literatures and entered in to EPI Info version 3.5.1. Bivariate analysis between dependent and independent variables was performed. Multivariate analysis was also done to control for possible confounding variable by selecting variable which show statistically significant association (P < 0.2) in bivariate analyses to identify independent predictor factors.

**Results:**

The proportion of community utilization of health extension service was 39%. Age (AOR = 2.52; 95% CI = 1.53-4.15), occupation (AOR = 3.79; 95% CI = 1.64-12.5), knowledge of community on health extension service (AOR = 0.25; 95% CI = 0.18-0.36), community participation in planning of health extension activities (AOR = 0.22; 95% CI = 0.15-0.33) and graduation of model family (AOR = 0.74; 95% CI = 0.47-0.76) have statistically significant association with community health extension services utilization.

**Conclusions:**

The proportion of community utilization of health extension service was low. Age, occupation, knowledge of community on health extension service, community participation in planning of health extension activities and graduation of model family were identified as the independent factors affecting the community’s utilization of health extension services.

## Background

### Background Information

Ethiopia is currently implementing twenty-years, rolling plans called Health Sector Development Plan (HSDP) which is divided in 3–5 years. The review of the first HSDP performance (1997/98- 2001/02) indicated the challenges of achieving universal coverage of Primary Health Care (PHC). It revealed that the necessary basic health services have not reached to the people at the grass roots level as predicted and desired [1]. In response to this, the Ethiopian government initiated the Health Extension Program in 2003 as part of the HSDP to improve equitable access to health service, preventive, promotion and selected curative health interventions through health extension workers [2].

The Health Extension Program (HEP) is an innovative community level component of the Essential Health Services Package (EHSP) of the country that was initially started as pilot program during the second HSDP (2002/03-2004/05) in 50, 17, 24 and 3 ‘kebeles’ (small administrative unit) of Tegray, Oromia, Amhara, SNNP (Southern Nation Nationality and people) and Dire Dawa regional state, respectively. Currently Technical and Vocational Training centers (TVETC) are being used to train health extension workers all over the country [3]. This can facilitate the achievement of health related millennium development goals in particular and other Millennium Development Goals (MDGs) in general [4].

The implementation of HEP involves deployment of two trained and salaried female HEWs at each ‘kebeles’. These health extension workers are posted to rural communities across Ethiopia, where they provide a better and more equitable access to health services for the poor, women, and children in a sustainable manner [5].

The program provides 16 different packages to reach the poor and address inequities and focusing on four areas of care: Disease Prevention and Control, Family Health, Hygiene and Environmental Sanitation, and Health Education and Communication. HEWs spend 75% of their time by visiting families in their homes and performing outreach activities in the community. The remaining 25% is spent by providing services at the health posts, including immunizations and contraceptives, among others. To address strong community demands for basic curative care, HEWs are trained to provide first aid and to treat malaria, dysentery, intestinal parasites and other ailments. In addition, they refer cases to the nearest health center when care that is more complicated is needed [6].

Each kebele has a health post that serves 5,000 people and functions as an operational center for a health extension worker. Five health posts and one health center works in collaboration and for the Primary Health Care Unit (PHCU) that serves 25,000 people [7].

### Statement of the problem

Ethiopian children and mothers have been facing several health problems due to poor access to modern health care facilities and lack of effective demand to utilize the available ones. Consequently, Ethiopia's records on child and maternal health have remained rather dismal for many decades. With only 26% and 5% of the pregnant women getting antenatal care and delivery care by trained health professional, respectively. Ethiopia has one of the lowest use of maternal health services in sub-Saharan Africa [8]. Because of restricted access to modern health care, over 676 maternal deaths occur for every 100,000 live births in the country and many of the pregnancies are at high risk. The infant mortality rate was 59 per 1,000 live births, the child mortality rate was 31 per 1,000 children surviving to age 1 year, and the under-five mortality rate was 88 per 1,000 live births. This implies that one in 17 Ethiopian children dies before the first birthday and one in 11 Ethiopian children dies before the fifth birthday [9].

Throughout the 1990s, poor nutritional status, infections, and a high fertility rate, together with low levels of access to basic health services in Ethiopia, contributed to one of the highest maternal and child mortality ratios in the world. In 2005, almost all births took place at home, with only 6 percent of women delivering in a clinic or hospital. Major causes of morbidity and mortality for children under five years of age were preventable. Extrapolations from the, 2005 demographic and health survey showed malnutrition to be the underlying cause of more than half of deaths of children under five [10].

In 2005, only 1% of households owned a bed net, of which less than 18% were insecticide treated. The brunt of poor health fell on the rural poor; most of them live out of reach of health providers. Only 40% of the population lived less than 10 kilometers from a clinic or other health service delivery point. Although more than 85% of the population lived in these rural areas, doctors and nurses preferred to work in urban hospital settings, where professional opportunities were better [8].

To improve the above-mentioned problems, Ethiopia has launched an innovative community-based health extension services program in 2003 at national level [2]. The primary purpose of the health extension services program is to improve access and utilization of preventive, promotive, and basic curative services especially for children’s and mother’s in the country by creating opportunities to enable households to exercise a health practice and living healthy through comprehensive, interrelated, economically and technically feasible health interventions [11,12].

The government of Ethiopia has made efforts in expanding health extension program to rural communities across the country. Even though there was a progress in intensifying the health extension program to rural communities, there is no evidence showing the gap between the health extension service provision and community health extension service utilization. Therefore, the purpose of this study was to identify the level of health extension service utilization and associated factors that help to develop targeted strategies for improving the health extension service provision and community’s health extension service utilization.

## Methods

### Study area and period

The study was carried out in Abuna Gindeberet district from February to March 2012. Abuna Gindeberet is one of the districts in west shoa zone of Oromia regional state. The administrative town of this district is located 176 KM from Addis Ababa. Based on the national census of 2007, the district had an estimated total population of 126,049 of whom 64,285 and 61,764 were males and females, respectively. The district has 43 ‘kebeles’ of which two urban and 41 rural ‘kebeles’. All the ‘kebeles’ have health extension workers. According to information from district health office, there were about 91 health extension workers and 51 other health workers rendering the health service to the community at large. The ratio of health extension workers to households was 1:289.

### Study design

A community based cross–sectional study was conducted on randomly selected heads of households who fulfilled the following inclusion criteria: (1) households living for more than one year in the study area and (2) households’ heads age of 18 years and older.

### Sample size determination

Sample size was determined by using single population proportion formula *n* = (*Zα*/2)^2^ × *P* (1 - *p*)/*d*^2^] with the assumption of 50% proportion since no previous study done, at 95% confidence interval, margin of error 5%, 10% none response rate and design effect 2. Finally, the required sample size was 806 head of Households.

### Sampling technique

The study had employed multistage sampling technique. By using simple random sampling technique, 10 from 41 rural ‘kebeles’ were selected and the sample size was distributed proportionally to the size of their households. By considering households registration numbers from registration book as a sampling frame, Systematic random sampling technique was employed to select households from selected kebeles. The study participants were selected every eight (8^th^) household intervals, by dividing the total number of households to the allocated sample size.

Revisit of two times was made in case where eligible respondents where not available by the time of the survey. In case where illegible subjects in the selected households where not available the next household was interviewed.

#### *Dependent variable*

Health extension service utilization.

#### *Independent variables*

For this study, the independent or predictor variables used were age, sex, marital status, religion, educational status, occupation and family size, knowledge of the community on health extension package, community involvement/participation in planning of health extension activities and graduation of model family. The detail descriptions of some variables were as follow;

Age refers to the respondents age at the time of the survey, has six categories ranging from ≤19, 20–24, 25–29, 30–34, 35–39 and Above 39 years. Educational status refers to the highest educational level the respondent attained and it was categorized as Illiterate, Read & write, 1–8, 9–12 and above grade 12. Religion was classified according to the previous literature as orthodox, protestant, Muslim and traditional religion. Occupation of the respondent has four categories like farmer, merchant, government employed and daily labors. Marital status were also classified as single, married, widowed, divorced and separated based on the answer from the respondents. Family Size refers to the number of family members the head of the household have during the survey time and has three category ≤5, 6–10 and above 10. The rest variables were described in the operational definition.

### Operational definition

#### *Knowledge of HEP*

Was measured based on respondent's ability to respond the questions related to health extension package. Respondent's score of below the mean (<50%) were classified as having unsatisfactory knowledge and those who score above or equal to the mean (≥50) were considered as having satisfactory knowledge on health extension package/services.

#### *Health extension service utilization*

Was measured using respondent's utilization of selected health extension services (services given by health extension workers at health post and outreach). Respondent's score above or equal to the mean were considered as utilized and respondent's score below the mean were classified as not utilized.

#### *Model families*

Are those households that have received theoretical and practical training on 16 health extension packages for at least three months or 94 hours to adopt healthy practices and serve as ‘models’ in their neighborhood.

### Data collection procedures

Data was collected through face-to-face interview by using pretested structured Afan Oromo version questionnaires adopted from review of different related literatures. The questionnaire was first prepared in English and translated to Afan Oromo and back to English to check its consistency by respective language experts. In addition, it was pretested and revised accordingly before undertaking the main study. Five supervisors who were diploma holder nurses and twelve completed data collectors with previous experience of data collection and fluent speaker of the local languages were recruited Additional file 1.

### Data quality control

To ensure data quality, adequate training was given to data collectors and supervisors to increase the reliability of the data collectors. During data collection, data collectors were supervised on how they are administering questions and randomly visiting household. The supervisors also checked all the filled questionnaires for completion, clarity and proper identification of the respondents every day. The principal investigator double-checks randomly for the completion each day. Incomplete and unclear questionnaires were returned to the subject (one who fill it) to get it completed for the next day using the codes given to the questionnaires and households during the data collection. After data collection was completed re-check-up was made in each kebeles before leaving the area. Finally, the data was cleaned thoroughly and double entered before analyses.

### Data processing and analysis

The data was first checked manually for completeness and entered to EpI Info version 3.5.1 computer software. The entered data was transferred to SPSS version 16 computer software program for further processing. Descriptive statistics such as proportions, percentages, means were used and data was presented with tables & texts.

Bivariate analysis was conducted primarily to check which variables have association with the dependent variables individually. Variables found to have association with the dependent variables at 0.2 probability were then entered in to multivariate logistic regression for controlling the possible effect of confounders and finally the variables which had significant association were identified on the basis of OR, with 95% CI and 0.05 p-values and fitted into the final model.

### Ethical considerations

The survey was conducted after the ethical clearance was obtained from research ethical committee of Haramaya University College of Medicine and Health Sciences. Informed written consent was obtained from respondents after explaining the objective of the study. They were informed that their participation was voluntary. Data was collected after assuring the confidential nature of responses and consent was obtained from the study participants.

## Results

From the total of 806 household's respondents, data was collected from 400 (49.6%) male and 406 (50.4%) female participants making the response rate of 100%. The age of household respondents ranges from 19–65 years, with the median age of 38 years. The majority, 305 (37.8%) of respondents’ age was found to be above age group of 39 year. Concerning educational status, half of the respondents 413 (51.2%) was not able to read and write. The distribution of respondents by religious affiliation showed that protestant were the dominant one 506 (62.8%) and 804 (99.8%) of them were belongs to Oromo ethnic group and speak Afaan Oromo (Table 1).

**Table 1:** Socio demographic characteristics of the respondents in Abuna Gindeberet District, Ethiopia, 2012

**Socio-demographic variables**	**Frequency (n = 806)**	**Percentage (%)**
***Age***		
≤ 19	11	1.4
20-24	69	8.6
25-29	153	19
30-34	125	15.5
35-39	143	17.7
Above 39 years	305	37.8
***Sex***		
Male	400	49.6
Female	406	50.4
***Educational status***		
Illiterate	413	51.2
Read & write	36	4.5
1-8	286	35.5
9-12	54	6.7
Above grade 12	17	2.1
***Marital status***		
Single	25	3.1
Married	718	89.1
Widowed/divorced/separated	63	7.8
***Religion***		
Orthodox	182	22.6
Protestant	506	62.8
Qallu	118	14.6
***Occupation***		
Farmer	740	91.8
Merchant	24	3.0
Governmental employed	15	1.9
Daily labors	27	3.3
***Family size***		
≤5	312	38.7
6-10	448	55.6
Above 10	46	5.7

### Knowledge of community on health extension services

To assess the community’s knowledge on health extension services, average (mean) scoring technique was used. Personal hygiene, healthy home environment, latrine construction and use, water supply and safety measure, antenatal care services, delivery service, family planning, immunization, essential nutrition action, HIV/AIDS prevention and control, STIs prevention and control, TB prevention and control, malaria prevention and control were the parameters used to generate mean.

Based on the mean scoring technique used, 335 (41.6%) of the respondents had satisfactory knowledge on health extension services by considering household respondents scoring greater than or equal to mean as having satisfactory knowledge. Specifically 350 (43.4%) of the study participants had knowledge about the benefit of latrine construction and use, 259 (32.1%) about family planning, 239 (29.7%) about Immunization, 172 (21.3%) about HIV/AIDS prevention and control where as few numbers of the respondents 96 (11.9%) have knowledge about prevention and control of STIs (Table 2).

**Table 2:** Knowledge of community on health extension services in Abuna Gindeberet District, Ethiopia, 2012

**Variables**	**Frequency (n = 806)**	**Percentage (%)**
**Knowledge of HEP/Services**		
Satisfactory	335	41.6
Unsatisfactory	471	58.4
**Type of HEP/Services they know**		
***Hygiene & Environmental sanitation***		
Personal hygiene	292	36.2
Healthy home environment	311	38.6
Latrine construction and use	350	43.4
Water supply and safety measure	245	30.4
***Family health services***		
Antenatal care services	114	14.1
Delivery service	95	11.8
Family planning	259	32.1
Immunization	239	29.7
ENA	158	19.6
***Disease prevention and control***		
HIV/AIDS prevention and control	172	21.3
STIs prevention and control	96	11.9
TB prevention and control	125	15.5
Malaria prevention and control	118	14.6

### Model family graduation and community participation in planning

From the household respondents who were asked whether they were graduated as model family, only 169 (21.0%) of respondents had reported as they were graduated as a model family of which 150 (88.8%) of them were reported as having certificate. The length of training shows a discrepancy accordingly, 70 (41.4%) of them received training for three months whereas 23 (13.6%) for six months, 21 (12.5%) for nine months and the rest 55 (32.5%) were received training for two weeks only. Concerning community participation, 265 (32.9%) of household respondents were participated in planning of health extension activities (Table 3).

**Table 3:** Community participation in planning and model family graduation in Abuna Gindeberet, Ethiopia, 2012

**Variables**	**Frequency (n = 806)**	**Percentage (%)**
**Participation in planning of HEP activities**		
Yes	265	32.9
No	541	67.1
**Graduation of model family**		
Yes	169	21.0
No	637	79.0
**Length of training**		
Two weeks	55	32.5
Three months	70	41.4
Six months	23	13.6
Nine months	6	3.6
One year	15	8.9
**Certificate seen**		
Yes	150	88.8
No	19	11.2

### Level of health extension services utilization

Community utilization of health extension service was measured using respondent's utilization of selected health extension services (services given by health extension workers at health post and outreach). Respondent's score above or equal to the mean were considered as utilized and respondent's score below the mean were classified as not utilized. Based on the above information, more than two third of study participants 314 (39%) had scored above the mean value which is considered as those who had utilized health extension services. Specifically 297 (36.8%) of the study participants where using latrine, whereas 226 (28%) have used family planning, 240 (29.8) have used child immunization and women immunization was used by 203 (25.2%) of the respondents. However, Antenatal care services, delivery services and postnatal care were services less utilized by 38 (4.7%), 23 (2.9%) and 36 (4.5%) of study participants, respectively (Table 4).

**Table 4:** Level of health extension service utilization in Abuna Gindeberet District, Ethiopia, 2012

**Variables**	**Frequency (n = 806)**	**Percentage (%)**
**Utilization of services**		
Utilized	314	39.0
Not utilized	492	61.0
**Type of HEP/services they used**		
Family planning services	226	28.0
Child immunization services	240	29.8
Women immunization service	203	25.2
Antenatal care services	38	4.7
Delivery services	23	2.9
Postnatal care services	36	4.5
Health education	143	17.7
Treatment services	90	11.2
Latrine construction and use	297	36.8
Waste disposal service	310	38.5
Community conversation	42	5.2
Model family training services	387	48.0
Healthy home environment	278	34.5

### Factors associated with community’s utilization of health extension service

Multiple logistic regression model revealed that: age, occupation, knowledge of community on health extension service, community participation in planning of health extension activities and graduation of model family were identified as independent factors affecting the community health extension service utilization.

Household respondents who have unsatisfactory knowledge on health extension service were 0.25 times less likely to utilize health extension services when compared to those respondents who have satisfactory knowledge (AOR = 0.25; 95% CI = 0.18-0.36). Household respondents who did not participated in planning of health extension activities were 0.22 times less likely to utilize health extension services when compared with those respondents who were participated in the planning process of health extension activities (AOR = 0.22; 95% CI = 0.15-0.33). In addition, household respondents who were not graduated as model family were 0.74 times less likely to utilize the health extension service as compared to those respondents who were graduated as model family (AOR = 0.74; 95% CI = 0.47-0.76) (Table 5).

**Table 5:** Multivariate logistic regression analysis of independent factors affecting health extension service utilization in Abuna Gindeberet District, Ethiopia, 2012

**Variables**	**Health extension service utilization**		**COR (95% CI)**	**AOR (95% CI)**
	**Utilized**	**Not utilized**		
***Age***				
≤19	3(27.3%)	8(72.7%)	0.77(0.2-2.96)	1.38(0.28-6.81)
20-24	16(23.2%	53(76.8%)	0.62(0.34-1.14)	0.59(0.28-1.25)
25-29	54(35.3%)	99(64.7%)	1.12(0.74-1.68)	1.09(0.66-1.81)
30-34	58(46.4%)	67(53.6%)	1.78(1.16-2.72)*	1.97(0.18-3.29)
35-39	83(58%)	60(42%)	2.84(1.88-4.27)*	2.52(1.53-4.15)**
Above 39 years	100(32.8%)	205(67.2%)	1.00	1.00
***Occupation***				
Farmer	279(37.7%)	461(62.3%)	1.00	1.00
Merchant	15(62.5%)	9(37.5%)	2.76(1.19-6.38)*	2.78(0.03-3.60)
Government employer	11(73.3%)	4(26.7%)	4.54(1.43-14.4)*	3.79(1.64-12.5)**
Daily labor	9(33.3%)	18(66.7%)	0.83(0.37-1.86)	1.28(0.43-3.79)
***Knowledge on HEP***				
Unsatisfactory	102(21.7%)	369(78.3%)	0.16(0.12-0.22)*	0.25(0.18-0.36)**
Satisfactory	212(63.3%)	123(36.7%)	1.00	1.00
***Participation in planning***				
No	134(24.8%)	407(75.2%)	0.16(0.11-0.220*	0.22(0.15-0.33)**
Yes	180(67.9%)	85(32.1%)	1.00	1.00
***Model family graduation***				
No	206(32.3%)	431(67.7%)	0.27(0.19-0.39)*	0.74(0.47-0.76)**
Yes	108(63.9%)	61(36.1%)	1.00	

*Significant association in bivariate.

**Significant association in multivariate.

## Discussion

The result of this study shows that the proportion of community utilization of health extension service was 39%. Especially maternal health care service like antenatal care services, delivery services and postnatal care services were among the service utilized by few study participants. In 2007, another study conducted by center for national health services on functioning of health posts support this finding. The study reported that most of health posts could provide family planning services; antenatal care and more than half could perform clean and safe deliveries and postnatal care. In contrast, the post-natal care and assisted delivery coverage remain low [13]. Similarly, recently completed third round health extension program evaluation also showed that few women in Ethiopia receive postnatal care and most of women still give birth at home [14].

The study conducted in Guba lafto district on practices of women during pregnancy and childbirth with the perspectives of health extension workers role also support this result, that only few study participant use antenatal care, delivery service and postnatal care [15]. The main underlying factor for low births attendance was low skill levels of health extension workers in assisting deliveries and to some extent the cultural barriers to deliver at the modern health facility [16].

In contrary to this finding, the study conducted by last ten-kilometer project founds that proportion of children received immunization, the use of any contraceptive method among currently married women and women inoculated against tetanus was high [14]. Similarly, the study conducted on impact evaluation of the Ethiopian health services in 2009 also founds that a significantly larger proportion of children were vaccinated against diphtheria, tetanus, measles, polio, tuberculosis and main antigens [17]. This may be due to lack of community basic understanding of health extension package as a result they were not able to change in practice what they know and suggested by health extension workers.

The proportion of community knowledge on health extension services was unsatisfactory (41.6%). This might be in rural community imparting health-promoting information in a non-standard environment, when mothers may have other demands were being placed upon them is without doubt a challenging task and given the well-established difficulties of communicating health information to poorly educated people. The finding of the study conducted on initial community perspectives on the health Service extension in Welkite revealed that low levels of community knowledge regarding the major communicable diseases [17].

Knowledge of community on health extension service was one of the factors that affect community utilization of health extension service. Household respondents who have unsatisfactory knowledge on health extension service were 0.25 times less likely to utilize health extension services when compared to those who have satisfactory knowledge on health extension package/services (OR = 0.25; 95% CI = 0.18-0.36).

Community's participation in the planning of health extension activities was 32.9%. The federal ministry of health report confirms the result that there is low community participation in the health extension program; there is no community entry and involvement in the planning process of the health extension program [3]. Community participation in planning of health extension activities also found to be independent factors affecting community utilization of health extension service. Households respondents who did not participated in planning of health extension activities were 0.22 time less likely to utilize health extension services when compared to those household who had participated in the planning process of health extension activities (OR = 0.22; 95% CI = 0.15-0.33). This may be explained by the fact that the greater the participation of the community, the greater the acceptance and use of services and the lesser the demand for expensive curative services [18]. If communities are involved in planning process, some of the complaints from the communities during peak farming periods will be avoided [19]. In addition, communities feel sense of ownership if they participated in the planning process of health extension activities.

The proportion of graduated model family was 21.0%. This result was high when compared to the finding of the studies conducted in 2009 in the four regions of Ethiopia. It was reported that only 4% of the respondents were graduated as a model family and half-presented certification to the interviewer [14]. Similar study conducted in the case of Southern Ethiopia also reported that 4.3% households were certified as model households [20]. Graduations of model family were statistically associated with utilization of health extension service. Household respondents who were not graduated as model family were 0.74 times less likely to utilize the health extension service as compared to those household respondents who were graduated as model family (OR = 0.74; 95% CI = 0.47-0.76). The possible explanations for this may be due to household’s have to go through a number of steps to graduate as a ‘model’ family. They receive training on sixteen (16) health extension packages for at least three months to adopt healthy practices and serve as ‘models’ in their neighborhood which helps them to have better understanding, knowledge and more likely to utilize the health extension services.

### Study limitations

This study provided important information regarding the level of community’s utilization of health extension services and assessed some of the factors that influence health extension service utilization among communities. Lack of previous studies conducted on this particular topic, being a cross sectional study and there may be social desirability and recall bias among study participants.

## Conclusions

The study provided important information regarding to the level of community utilization of health extension service and associated factors. The proportion of community’s utilization of health extension service was low. Age, occupation, knowledge of community on health extension service, community participation in planning of health extension activities and graduation of model family were identified as the independent factors affecting community health extension services utilization.

Thus, district health office should create-intensified awareness creation targeted to increase community understanding of health extension package in order to bring behavioral change, involve community in planning of health extension activities to increase community participation, create-intensified training on model family to increase health extension service utilization in the community. Further research should be undertaken to determine factors affecting utilization of health extension service from the perspective of health extension workers on a wider scale.

### Endnotes

^a^***Knowledge of HEP*** was measured based on respondent's ability to respond the questions related to health extension package. Respondent's score of below the mean (<50%) were classified as having unsatisfactory knowledge and those who score above or equal to the mean (≥50) were considered as having satisfactory knowledge on health extension package/services.

^b^***Health extension service utilization*** was measured using respondent's utilization of selected health extension services (services given by health extension workers at health post and outreach). Respondent's score above or equal to the mean were considered as utilized and respondent's score below the mean were classified as not utilized.

^***c***^***Model families are those*** households that have received theoretical and practical training on 16 health extension packages for at least three months or 94 hours to adopt healthy practices and serve as ‘models’ in their neighborhood.

## Competing interest

The authors declare that they have no competing interests.

## Authors’ contributions

Zk conceived the study, participated in the data collection, analysis, interpretation of the data and drafted the manuscript. NB and GE have made substantial contribution by editing and revising it critically for important intellectual content from the beginning. All authors have read and approved the final manuscript.

## Authors’ information

Zk is an MPH, in department of communicable disease prevention and control at zonal health office, Ambo, Ethiopia. NB is an MPH, Associate professor in Haramaya University, College of Medicine and Health Science, School of Graduate Studies, Harar, Ethiopia. GE is an MPH and PHD candidate in Haramaya University, College of Medicine and Health Science, School of Graduate Studies, Harar, Ethiopia.

## Pre-publication history

The pre-publication history for this paper can be accessed here:

http://www.biomedcentral.com/1472-6963/14/324/prepub

## Supplementary Material

Additional file 1 Questionnaires used to asses level of health extension service utilization and associated factors among community in Abuna Gindeberet District, Ethiopia, 2012. Click here for file
