# Supplementary material for: Level of health extension service utilization and associated factors among community in Abuna Gindeberet District, West Shoa Zone, Oromia Regional State, Ethiopia
Source: BMC Health Serv Res. 2014 Jul 28;14:324. doi: 10.1186/1472-6963-14-324 (PMC4236544; doi:10.1186/1472-6963-14-324)
Supplement: Additional file 1 — Questionnaires used to asses level of health extension service utilization and associated factors among community in Abuna Gindeberet District, Ethiopia, 2012. [file 1472-6963-14-324-S1.doc]

### Appendix

### Appendix 1: Questionnaires used to asses level of health extension service utilization and associated factors among community in Abuna Gindeberet District, Ethiopia, 2012

Questionnaire Code______________________

Kebele _____________________________

House number________________________

| **Part I. Socio-demographic Characteristics of the respondents** | | | | | |
| --- | --- | --- | --- | --- | --- |
| No | Question | | | Response | Skip |
| 101 | Age in years | | | 1. _______________ years |  |
| 102 | Sex | | | 1.Male 2.Female |  |
| 103 | What is your level of education? | | | 1. Illiterate 2. read and write 3. 1- 8 grade 4. 9-12 grade 5. Above grade 12 |  |
| 104 | Which language do you speak? | | | 1. Afan Oromo language 2. Amharic language   99. Other Specify____________________ |  |
| 105 | What is your religion? | | | 1. Orthodox 2. Protestant 3. Muslim   99. Other Specify __________________ |  |
| 106 | What is your current marital status? | | | 1. Single 2. Married 3. Separated 4. Divorced 5. Widowed |  |
| 107 | To which ethnic group do you belong? | | | 1. Oromo 2. Amahara   99. Other Specify____________________ |  |
| 108 | What is your job (work) currently? | | | 1. Farmer 2. House wife 3. Merchant 4. Governmental employ   99. Other Specify____________________ |  |
| 109 | How many family members do you have? | | | ___________________________. |  |
| **Part II. Question on knowledge of community on Health Extension package** | | | | | |
| 201 | Did the health extension worker visit your family for the last 6 month? | | | 1. No 2. Yes |  |
| 202 | Do you know the benefit of health extension package/service listed below? | | | 1. About antenatal care   0.No 1.Yes   1. About pregnancy care   0.No 1.Yes   1. About family planning   0.No 1.Yes   1. About immunization   0.No 1.Yes   1. About nutrition   0.No 1.Yes   1. About environmental sanitation   0.No 1.Yes   1. About personal hygiene   0.No 1.Yes   1. About HIV/AIDS prevention and control 0.No 1.Yes 2. STIs prevention and control   0.No 1.Yes   1. About TB prevention and control   0.No 1.Yes   1. About malaria prevention and control   0.No 1.Yes   1. About clean house   0.No 1.Yes   1. Promotion pit latrine construction   0.No 1.Yes   1. Promote latrine use   0.No 1.Yes   1. Promote safe water use   0.No 1.Yes   1. About model family   0.No 1.Yes |  |
| **Part III. Questionnaires’ used to assess graduated model family in the community** | | | | | |
| 301 | Have you graduated as a Model family? | | 1. No 2. Yes | | 401 |
| 302 | For how long do you receive training on model family? | | 1. Three months 2. Six months 3. Nine months 4. One year   99. Other specify-------------------------- | |  |
| 303 | Do you have certificate? | | 1. No 2. Yes | |  |
| **Part V. Questionnaires to assess community participation in health extension activities** | | | | | |
| 401 | Do you participate in the implementation of health extension activities? | | 1. No 2. Yes | |  |
| 402 | Do you participate in the planning of health extension activities? | | 1. No 2. Yes | |  |
| 403 | Do you participate in the health post construction? | | 1. No 2. Yes | |  |
| **Part V. Questionnaires to assess community health extension services utilization** | | | | | |
| 501 | DO you use health extension package/services listed below? | 1. Family planning service 0.No 1.Yes 2. Child immunization services 0.No 1.Yes 3. Women immunization services 0.No 1.Yes 4. Antenatal care services 0.No 1.Yes 5. Delivery services 0.No 1.Yes 6. Postnatal care services 0.No 1.Yes 7. Health education service 0.No 1.Yes 8. Treatment services 0.No 1.Yes 9. Latrine construction and use 0.No 1.Yes 10. Waste disposal service 0.No 1.Yes 11. Community conversation 0.No 1.Yes 12. Model family training services 0.No 1.Yes 13. Healthy home environment 0. No 1. Yes | | |  |
